# Optimizing [^18^F]FAPI-74 PET/CT imaging: insights from Early-phase tumor uptake and tissue contrast

**DOI:** 10.1186/s13550-025-01356-x

**Published:** 2025-12-04

**Authors:** Yuki Hoda, Keiji Shimizu, Hiroyuki Nishida, Yasuhiko Ikari, Go Akamatsu, Keiichi Matsumoto, Michio Senda, Tomohiko Yamane

**Affiliations:** 1 Department of Molecular Imaging Research, Kobe City Medical Center General Hospital, Minatojima-Minamimachi 2-1-1, Chuo-ku, Kobe, 650-0047 Japan; 2 Department of Diagnostic and Interventional Radiology, Nara Medical University, Kashihara, Japan; 3 Department of Radiological Technology, Kobe City Medical Center General Hospital, Kobe, Japan; 4 National Institutes for Quantum Science and Technology, Chiba, Japan; 5 Department of Biomedical Information Sciences, Hiroshima City University, Hiroshima, Japan

**Keywords:** Fibroblast activation protein inhibitor, [^18^F]FAPI-74, PET/CT, Early-phase imaging, Cancer-associated fibroblast

## Abstract

**Background:**

Fibroblast activation protein inhibitor (FAPI) is a novel PET tracer that targets various malignant tumors. Although some studies have demonstrated that ^68^Ga-labeled FAPI PET provides diagnostic images shortly after injection, reports on early-phase imaging using [^18^F]FAPI-74 are limited. This study evaluated the diagnostic potential of early-phase [^18^F]FAPI-74 PET/CT imaging.

**Methods:**

The cohort comprised 32 patients who underwent whole-body [^18^F]FAPI-74 PET/CT at three time points: 10, 30, and 60 min after administration. The uptake of [^18^F]FAPI-74 in normal organs and tumors at each time point was evaluated and compared using standardized uptake value (SUV). The contrast between tumor and background uptake was assessed using tumor-to-blood ratios (TBRs), calculated as the ratio of tumor SUVmax to blood pool SUVmean.

**Results:**

The uptake of [^18^F]FAPI-74 in most organs gradually decreased, except in the mammary gland and nipple. Moreover, uptake due to the excretion increased over time in the gallbladder, and bile duct. Among the 72 lesions identified across patients, the median SUVmax of the tumors remained relatively stable across the time points (5.00, 5.16, and 5.11 at 10, 30, and 60 min, respectively), while the median TBRs gradually increased (1.57, 2.24, and 2.94, respectively). Subgroup analysis revealed a similar pattern of increasing median TBRs in primary tumors (2.76, 3.72, and 4.04, respectively), lymph node metastases and peritoneal dissemination (1.67, 2.47, and 3.58, respectively), and lung metastases (0.99, 1.35, and 1.48, respectively).

**Conclusions:**

TBRs were highest at 60 min after administration. However, early-phase imaging was considered useful because of its sufficient diagnostic contrast and low physiological uptake in the biliary system.

**Trial registration:**

UMIN Clinical Trials Registry, UMIN000051687. Registered 22 July 2023, https://center6.umin.ac.jp/cgi-open-bin/ctr_e/ctr_his_list.cgi?recptno=R000058594.

## Background

The tumor microenvironment, which surrounds tumor cells, plays a crucial role in tumor progression. It is composed of various non-tumor cells—including fibroblasts, vascular cells, and immune cells—as well as the extracellular matrix. Among these, cancer-associated fibroblasts (CAFs) are a key component of the tumor microenvironment. They secrete multiple growth and angiogenic factors and contribute to tumor progression, invasion, metastasis, immunosurveillance, and drug resistance. CAFs highly express fibroblast activation protein (FAP), which is found in more than 90% of epithelial tumors [1]. FAP is a type II membrane-bound glycoprotein, and its expression is associated with poor prognosis in several types of cancer [2]. Because of its abundant and selective expression in the tumor stroma, FAP has gained increasing interest as a target for molecular imaging and therapy. Accordingly, FAP inhibitor (FAPI) has been developed, and FAP-based PET tracer have shown promise for imaging a wide range of malignant tumors [1, 3, 4].

In the early stages of clinical use, ^68^Ga-labeled FAPI compounds were predominantly employed due to their ease of synthesis [5–7]. Previous studies on the optimal imaging times for ^68^Ga-labeled FAPI PET/CT have shown that while contrast improves over time, early-phase imaging still provides sufficient detectability [3, 8–16]. Naemi et al. recommended 10–20 min after administration [8], whereas Glatting et al. recommended early-phase imaging at 30–40 min after administration as the optimal timing [9].

However, ^68^Ga-labeled FAPI has several limitations. These include its short physical half-life, relatively high positron energy—which results in a longer positron range and consequently reduced special resolution—and the limited production capacity of ^68^Ge/^68^Ga generators, which are costly and restrict the amount of radiotracer that can be produced per batch. To overcome these limitations, ^18^F-labeled FAPI compounds have been developed following recent advances in radiochemistry [17, 18].

Most [^18^F]FAPI-74 PET/CT studies have performed imaging 60 min after administration [19–22], and reports focusing on early-phase imaging remain limited. Nevertheless, a few studies have investigated early-phase imaging protocols using ^18^F-labeled FAPI. Hu et al. used [^18^F]FAPI-42 to perform dynamic imaging for 20 min and static imaging at 1 and 2 h, showing that tumor uptake increased at 18 min and remained constant for up to 2 h, and reported that the optimal image acquisition time was 1 h after injection [23]. Mu et al. conducted two time point imaging with [^18^F]FAPI-42 PET/CT and reported no significant difference in diagnostic accuracy between the early and late phases, although contrast was enhanced in the later phase [24]. Giesel et al. imaged PET/CT at 10 min, 1 h, and 3 h after [^18^F]FAPI-74 injection and reported that the tumor-to-blood ratio (TBR) was most elevated at 1 h [25].

The diagnostic capability using early-phase imaging is expected to reduce patient burden and improve throughput in examination scheduling. However, if diagnostic performance declines, these benefits would be negated. Therefore, it is essential to characterize imaging features in the early-phase and identify appropriate clinical applications. Accordingly, this study aimed to evaluate the diagnostic potential of early-phase [^18^F]FAPI-74 PET/CT imaging.

## Materials and methods

### Patients

A study evaluating the pathophysiology of malignant tumors using [^18^F]FAPI-74 PET/CT was registered in the UMIN Clinical Trials Registry (UMIN000051687). The inclusion criteria for this study were as follows: individuals aged ≥ 18 years at the time of consent acquisition, those diagnosed with or strongly suspected of having a malignant tumor, those who provided consent for [^18^F]FAPI-74 results to be used in clinical decision-making if deemed necessary by their primary physician, and those capable of providing written informed consent. The exclusion criteria were pregnancy or the possibility of pregnancy, severe claustrophobia, and cases in which the principal investigator or co-investigator deemed participation in the study inappropriate.

From the original study cohort, cases that underwent three-phase [^18^F]FAPI-74 PET/CT between August 2023 and December 2024 were included in the present analysis, resulting in a total of 32 patients. The diagnosis of malignancies and metastatic lesions was made by a board-certified nuclear medicine physician in conjunction with CT and other imaging findings or confirmed by imaging follow-up demonstrating treatment response. Histopathological confirmation was not available for all lesions.

The study was conducted in accordance with the principles of the Declaration of Helsinki. Ethical approval was obtained from the Ethics Committee of Kobe City Medical Center General Hospital (#23073). Informed consent was obtained from all participants.

### PET/CT scanning

[^18^F]FAPI-74 was synthesized using CFN-MPS200 (Sumitomo Heavy Industries, Tokyo, Japan) according to the method described by Naka et al. [26].

No specific preparation was required for any patient on the day of [^18^F]FAPI-74 PET/CT scanning. Imaging was performed using a Discovery 690 PET/CT scanner (GE Healthcare).

Following intravenous injection of [^18^F]FAPI-74 (mean dose, 240.59 MBq; range, 212.24–257.88 MBq), image acquisition was initiated according to a predefined protocol. The injection time was precisely recorded, and subsequent scans were started at exact post-injection intervals of 10, 30, and 60 min.

Within the first 10 min after administration, a non-contrast-enhanced low-dose CT scan was performed for attenuation correction using the following parameters: tube voltage, 120 keV; tube current, auto mA (range, 15–60 mA); and slice thickness, 3.75 mm. PET images were acquired from the middle of the thigh to the top of the head in a three-dimensional mode (matrix, 192 × 192) with an acquisition time of 2 min/bed position.

After the CT, the first (10 min) and second (30 min) PET scans were sequentially acquired. After urination, another low-dose CT scan was performed, followed by a third (60 min) PET scan. Each whole-body PET acquisition was performed with an acquisition time of 2 min per bed position. Depending on patient height, 7 to 9 bed positions were required to cover the area from the mid-thigh to the top of the head, resulting in a total scan time of approximately 14 to 18 min per acquisition.

### Image analysis

We used Volume Viewer Version 17.0 on AW VolumeShare 7 (GE Healthcare) for image analysis. Spherical volumes of interest (VOIs) were manually placed, and standardized uptake values (SUVs) were measured at each time point for tumor lesions and normal organs, including the brain, parotid gland, submandibular gland, thyroid, lung, mammary gland, nipple, liver, gallbladder, bile duct, spleen, pancreas, kidney, uterus, prostate, testis, muscle, bone, and blood pool. In male patients, the VOIs were not placed in the uterus, whereas in female patients, they were not placed in the prostate or testis. Additionally, when the structures of the mammary gland could be confirmed by CT, a VOI was placed on the breast, regardless of the sex.

VOIs with a diameter of 1.5–2 cm were placed in the parotid gland, submandibular gland, thyroid, mammary gland, nipple, bile duct, pancreas, kidney, uterus, prostate, and testis; 2–3 cm in diameter in the liver, spleen, bone, and blood pool; and > 3 cm in diameter in the brain, lung, and muscle to ensure the inclusion of each organ’s uptake while avoiding overlap with adjacent structures. When defining the VOI for the liver, all three phases were reviewed to ensure that the selected peripheral region did not include the uptake suspected to originate from the intrahepatic bile duct. Similarly, for the kidneys, all three phases were reviewed to avoid including the renal pelvis or ureter in the VOI. For bone uptake, the VOI was placed in the fourth or fifth lumbar vertebra. For blood pool measurements, the VOI was placed in the left ventricular cavity.

Uptake in normal organs was evaluated using SUVmax for the nipple, gallbladder, and bile duct and SUVmean for other organs. SUVmean was not used for the nipple and bile duct because their small size made it challenging to place the VOIs without spillover into the surrounding tissue, and uptake in the biliary system showed significant temporal changes and spatial non-uniformity.

Furthermore, uptake of tumor lesions was evaluated using SUVmax. Since large tumor lesions often show heterogeneous uptake due to necrosis or degeneration, making SUVmax a more reliable parameter than SUVmean. TBRs were calculated as the ratio of the tumor SUVmax of the hottest tumor lesion to the SUVmean of the blood pool.

### Statistical analyses

All statistical evaluations were conducted using EZR version 1.68 (Saitama Medical Center, Jichi Medical University, Saitama, Japan) [27], which is a modified version of R Commander. The Friedman test was used for comparisons between the three groups, and a multiple comparison test with Bonferroni correction was used for comparisons between each pair of groups. *p* < 0.05 was considered statistically significant.

## Results

**Fig. 1:**
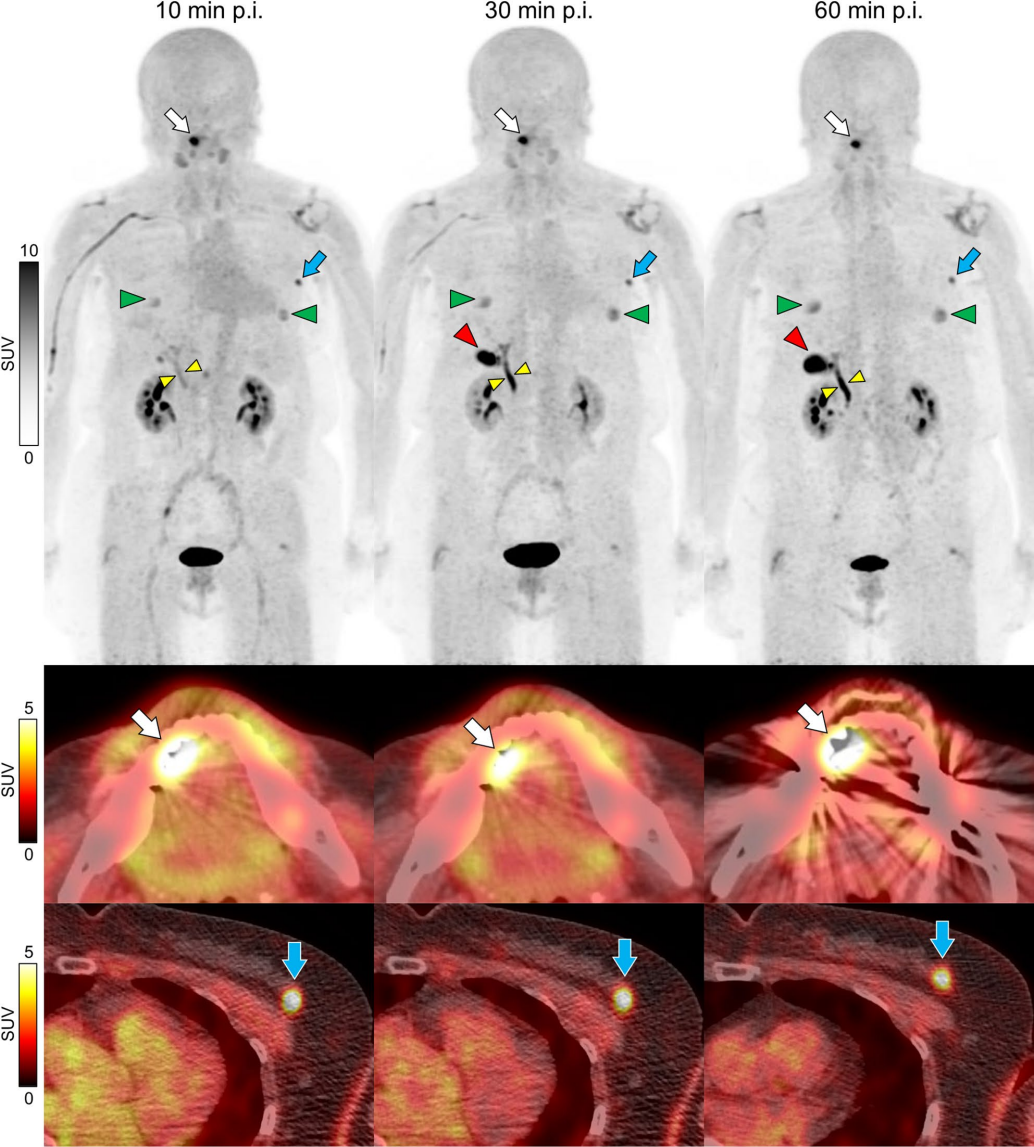
Representative case of three time point [^18^F]FAPI-74 PET/CT imaging Maximum intensity projection (MIP) images and axial [^18^F]FAPI-74 PET/CT fused images of 70-year-old female with tongue and breast cancers. Uptake in most normal organs such as the liver, spleen, and blood pool decreased over time. Uptake in the gallbladder (red arrowheads), bile duct (yellow arrowheads), and nipple (green arrowheads) gradually increased. There was an uptake of tongue cancer on the right side of the oral cavity (white arrows). Uptake in the left breast was incidentally detected in breast cancer (blue arrows). Grayscale indicates standardized uptake values (SUV) of the images. p.i.: post injection

Patient characteristics are presented in Table 1. The uptake of [^18^F]FAPI-74 in most organs decreased gradually over time. However, uptake increased in specific organs, including the mammary gland (SUVmean at 10, 30, and 60 min: 0.67, 0.81, and 0.94, respectively), nipple (SUVmax: 1.37, 1.63, and 1.87, respectively), gallbladder (SUVmax: 2.59, 8.95, and 11.51, respectively), and bile duct (SUVmax: 4.12, 8.65, and 9.83, respectively). In the uterus, uptake remained stable across over time (SUVmax: 2.73, 2.55, and 2.90, respectively). Representative maximum intensity projection images are shown in Fig. 1. The uptake in most normal organs decreased over time, except for gallbladder, bile duct, nipple. The uptake in tongue cancer (SUVmax: 16.12, 17.26, and 16.11, respectively) and breast cancer (SUVmax: 7.96, 7.30, and 6.80, respectively) remained relatively stable over time, and these cancers were clearly distinguishable from the early-phase.

**Table 1:** Patient characteristics

Characteristics	Data (Initial/Recurrence or follow-up)
Number of patients	32
Sex	
Male	19 (59.4%)
Female	13 (40.6%)
Age range (years)	26–86 (Median, 71.5)
Underlying diagnosis	
Head and neck cancer	7 (4/3)
Colorectal cancer	6 (0/6)
Gastric cancer	3 (1/2)
Esophagus cancer	3 (1/2)
Breast cancer	3 (1/2)
Gallbladder cancer	2 (1/1)
Hepatocellular cancer	1 (0/1)
Cholangiocarcinoma	1 (1/0)
Pancreatic cancer	1 (0/1)
Lung cancer	1 (1/0)
Cervical cancer	1 (0/1)
Bladder cancer	1 (0/1)
Mycosis fungoides	1 (0/1)
Benign lesions *	2

*Cases of benign lesions included a final diagnosis of a lateral neck cyst that was initially suspected to be a malignant neck tumor and a cystic mass in the mammary gland without obvious malignant findings on biopsy

One patient with multiple cancers (tongue and breast) was included in the study

**Fig. 2:**
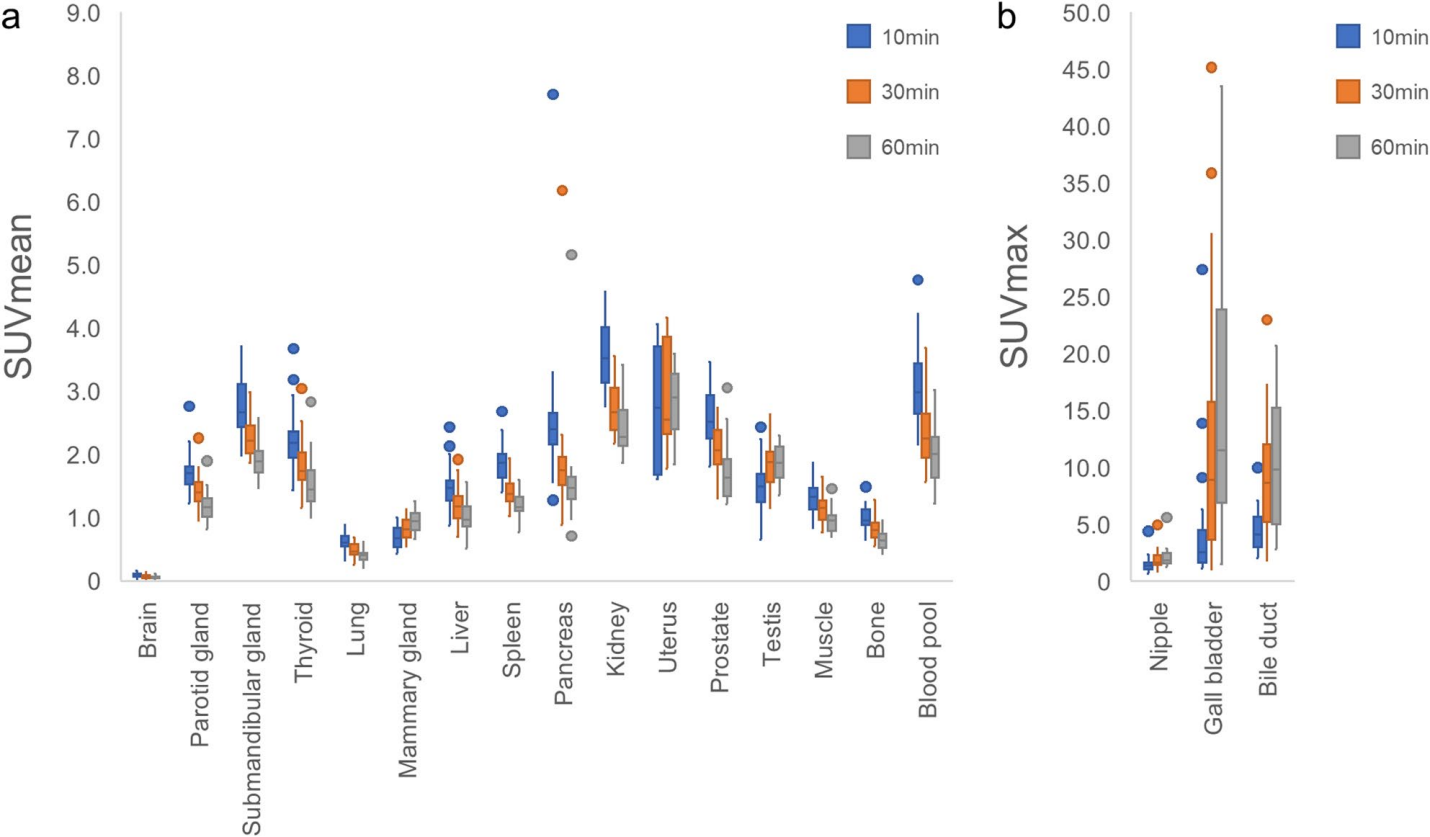
Uptake change of [^18^F]FAPI-74 in normal organs SUVmean (**a**) and SUVmax (**b**) are shown. Uptake in the organs gradually decreased, except in the mammary glands, nipple, gallbladder, bile duct, and uterus

The changes in the SUV over time are illustrated in Fig. 2, and the statistical data, including comparative analyses, are summarized in Table 2.

**Table 2:** [^18^F]FAPI-74 uptake and statistics in normal organs at three time points

Organ	*n*	Analyzed item	Median value			Friedman *p*-value	Bonferroni *p*-value		
			10 min	30 min	60 min		10 min vs. 30 min	30 min vs. 60 min	10 min vs. 60 min
Brain	32	SUVmean	0.09	0.06	0.06	< 0.01	< 0.01	0.15	< 0.01
Parotid gland	32	SUVmean	1.70	1.40	1.17	< 0.01	< 0.01	< 0.01	< 0.01
Submandibular gland	32	SUVmean	2.67	2.22	1.90	< 0.01	< 0.01	< 0.01	< 0.01
Thyroid	32	SUVmean	2.18	1.75	1.45	< 0.01	< 0.01	< 0.01	< 0.01
Lung	32	SUVmean	0.60	0.47	0.40	< 0.01	< 0.01	< 0.01	< 0.01
Mammary gland	14	SUVmean	0.67	0.81	0.94	< 0.01	< 0.01	0.07	< 0.01
Liver	32	SUVmean	1.47	1.18	0.97	< 0.01	< 0.01	< 0.01	< 0.01
Spleen	32	SUVmean	1.87	1.38	1.17	< 0.01	< 0.01	< 0.01	< 0.01
Pancreas	32	SUVmean	2.40	1.75	1.47	< 0.01	< 0.01	< 0.01	< 0.01
Kidney	32	SUVmean	3.52	2.67	2.28	< 0.01	< 0.01	< 0.01	< 0.01
Uterus	7	SUVmean	2.73	2.55	2.90	0.16	0.66	1	0.89
Prostate	19	SUVmean	2.51	2.07	1.63	< 0.01	< 0.01	< 0.01	< 0.01
Testis	18	SUVmean	1.50	1.88	1.87	< 0.01	< 0.01	1.00	< 0.01
Muscle	32	SUVmean	1.34	1.16	0.95	< 0.01	< 0.01	< 0.01	< 0.01
Bone	32	SUVmean	0.96	0.80	0.64	< 0.01	< 0.01	< 0.01	< 0.01
Blood pool	32	SUVmean	2.99	2.25	2.01	< 0.01	< 0.01	< 0.01	< 0.01
Nipple	32	SUVmax	1.37	1.63	1.87	< 0.01	< 0.01	< 0.01	< 0.01
Gallbladder	28	SUVmax	2.59	8.95	11.51	< 0.01	< 0.01	< 0.01	< 0.01
Bile duct	31	SUVmax	4.12	8.65	9.83	< 0.01	< 0.01	0.11	< 0.01

**Fig. 3:**
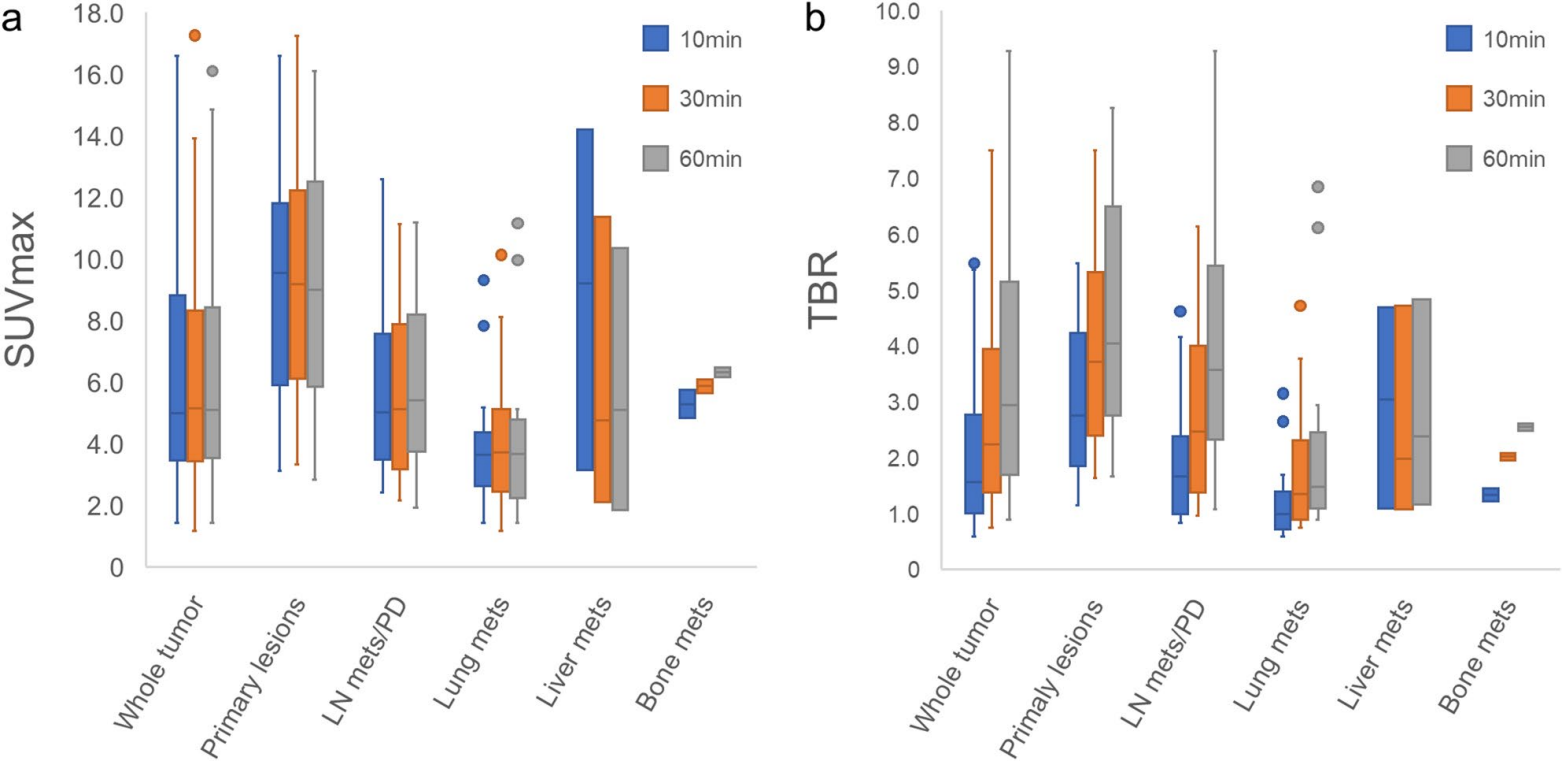
Change in [^18^F]FAPI-74 uptake in tumors While the SUVmax of the tumors remained relatively stable over time (**a**), tumor-to-blood ratios (TBRs) gradually increased (**b**). Subgroup analysis revealed a similar trend of increasing TBRs in primary tumors, lymph node metastases/peritoneal dissemination, and lung metastases. Mets: metastasis, LN: lymph node, PD: peritoneal dissemination

We identified 72 tumor lesions in 32 patients: 16 primary lesions (two each for breast cancer and gastric cancer, and one each for nasal and paranasal sinus cancer, tongue cancer, gingival cancer, nasopharyngeal cancer, oropharyngeal cancer, lung cancer, esophageal cancer, colon cancer, hepatic cell cancer, gallbladder cancer, cholangiocarcinoma, and pancreatic cancer), 32 lymph node metastases/peritoneal dissemination, 19 lung metastases, 3 liver metastases, and 2 bone metastases. The SUVmax of the whole tumor, including primary and metastases remained relatively stable across the time points (5.00, 5.16, and 5.11, respectively), whereas the TBRs gradually increased (1.57, 2.24, and 2.94, respectively). Subgroup analysis revealed a similar pattern of increasing TBRs in primary tumors (2.76, 3.72, and 4.04, respectively), lymph node metastases/peritoneal dissemination (1.67, 2.47, and 3.58, respectively), and lung metastases (0.99, 1.35, and 1.48, respectively). The changes in SUV and TBR over time are illustrated in Fig. 3, and the statistical data, including comparative analyses, are summarized in Table 3.

**Table 3:** [^18^F]FAPI-74 uptake and statistics in malignant lesions at three time points

Tumor/metastases	n	Analyzed item	Median value			Friedman *p*-value	Bonferroni *p*-value		
			10 min	30 min	60 min		10 min vs. 30 min	30 min vs. 60 min	10 min vs. 60 min
Whole tumor including primary and metastases	72	SUVmax	5.00	5.16	5.11	0.48	1	1	1
		SUVmean	3.20	3.22	3.29	0.38	1	0.51	1
		TBR	1.57	2.24	2.94	< 0.01	< 0.01	< 0.01	< 0.01
Primary tumors	16	SUVmax	9.56	9.19	9.01	0.44	1	1	0.89
		SUVmean	5.41	5.00	4.91	0.26	1	0.16	1
		TBR	2.76	3.72	4.04	< 0.01	< 0.01	< 0.01	< 0.01
Lymph node metastases/peritoneal dissemination	32	SUVmax	5.04	5.13	5.42	0.26	1	1	0.88
		SUVmean	3.20	2.99	3.29	0.93	0.82	1	1
		TBR	1.67	2.47	3.58	< 0.01	< 0.01	< 0.01	< 0.01
Lung metastases	19	SUVmax	3.65	3.73	3.68	0.34	0.23	1	1
		SUVmean	2.32	2.37	2.42	0.38	0.55	1	1
		TBR	0.99	1.35	1.48	< 0.01	< 0.01	< 0.01	< 0.01
Liver metastases	3	SUVmax	9.20	4.77	5.10	0.10	0.75	1	0.75
		SUVmean	5.45	2.74	3.00	0.10	0.75	1	0.75
		TBR	3.04	1.98	2.38	0.26	1	0.75	1
Bone metastases	2	SUVmax	5.29	5.88	6.33	0.14	1	1	1
		SUVmean	3.32	3.50	3.70	0.14	1	1	1
		TBR	1.34	2.02	2.55	0.14	1	1	1

TBR: tumor-to-blood ratio

**Fig. 4:**
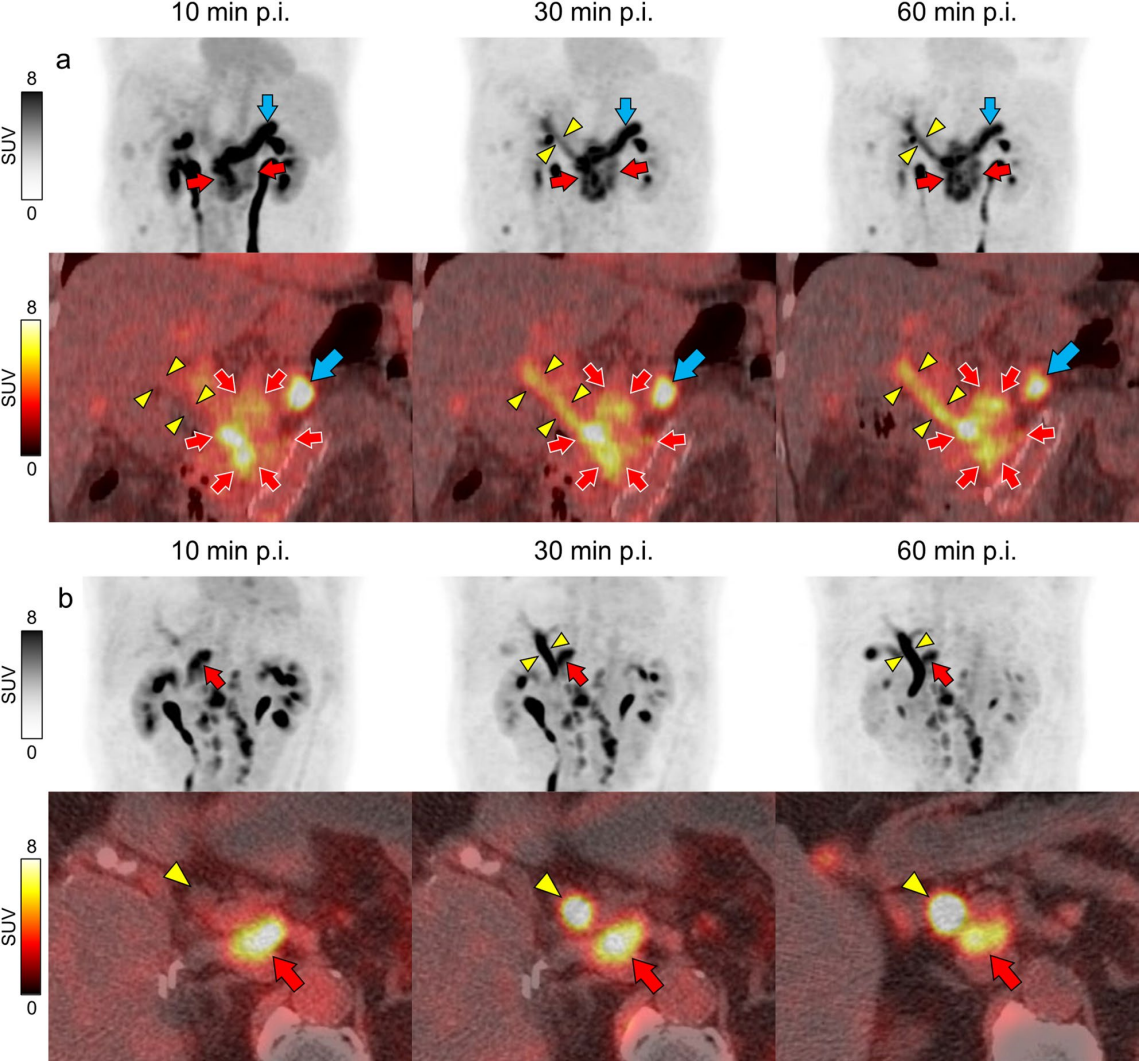
Representative cases of lesions located near the biliary system (**a**) MIP images and coronal [^18^F]FAPI-74 PET/CT fused images of a 62-year-old female with pancreatic head cancer. The tumor (red arrows) infiltrated the bile duct (yellow arrowheads), and uptake in the bile ducts increased over time. Increased uptake is observed in the pancreatic tail (blue arrow), suggesting fibrosis associated with chronic inflammation (**b**) MIP images and axial [^18^F]FAPI-74 PET/CT fused images of an 80-year-old male with para-aortic lymph node metastasis after surgery for ascending colon cancer. The metastatic lymph node (red arrow) is present on the dorsal aspect of the bile duct (yellow arrowhead), with increasing biliary tract uptake over time. Grayscales and color scales indicate standardized uptake values (SUV). MIP: Maximum intensity projection, p.i.: post injection

Two cases involved lesions located near the biliary system: one was a primary pancreatic head cancer, and the other was a para-aortic lymph node metastasis after surgery for ascending colon cancer (Fig. 4). Tumor uptake was relatively stable over time (SUVmax: 12.01, 10.08, and 10.91 in Figs. 4a and 9.84, 9,45, and 8.20 in Fig. 4b, respectively) and TBR was higher in late phase (4.20, 10.08, and 10.91 in Fig. 4a.07, 5.69, and 5.43 in Fig. 4b, respectively). The uptake in bile duct increased over time due to tracer excretion (SUVmax: 2.99, 6.49, and 7.52 in Figs. 4a and 5.50, 17.23, and 20.01 in Fig. 4b, respectively). Consequently, the visibility of these lesions decreased over time, as the tracer was excreted into the biliary system.

## Discussion

In this study, we compared the biodistribution and uptake of [^18^F]FAPI-74 in normal organs and tumor lesions at 10, 30, and 60 min after administration. The uptake of [^18^F]FAPI-74 in most organs gradually decreased over time, except in the mammary glands, nipples, gallbladder, and bile duct.

FAPI PET/CT is considered to be useful for the detection of breast cancer [28, 29]. However, because uptake in the mammary glands and nipples increased over time, imaging timing should be carefully considered to avoid potential misinterpretation. Uterine uptake also showed an increasing trend, although the difference was not statistically significant. Wang et al. reported that uptake in the uterus increased over time using dynamic imaging with [^68^Ga]FAPI-04 PET/CT [11]. Dendl et al. also reported that uptake in the endometrium and mammary gland was higher in premenopausal patients than in postmenopausal patients, suggesting that FAP expression in hormone-responsive organs may be influenced by hormone stimulation [28, 30]. Although menstrual history was not collected in this study, the uptake of [^18^F]FAPI-74 in hormone-responsive organs is likely to increase over time.

Additionally, uptake due to excretion into the biliary system increased over time. Compared with ^68^Ga-labeled FAPI, ^18^F-labeled FAPI is known to accumulate in the biliary system because of excretion. This is thought to be due to the use of NOTA as a chelating agent, which increases its lipophilicity [23, 24]. This may limit the detection of hepatobiliary or gastrointestinal lesions by [^18^F]FAPI PET/CT. Dai et al. reported that fat ingestion after [^18^F]FAPI-04 administration caused gallbladder contractions and decreased physiological [^18^F]FAPI-04 uptake, suggesting the possibility of improved diagnostic accuracy for gallbladder cancer [31]. In this study, the contrast between the two lesions near the biliary system decreased over time due to the biliary excretion of [^18^F]FAPI-74, making it difficult to visually separate them from the biliary system. In addition to fat intake, early-phase imaging may be useful for improving the visualization of lesions near the biliary system.

Blood pool uptake was relatively high, with an SUVmean of 2.99 at 10 min and 2.01 at 60 min, which was higher than that previously reported for other FAPI tracers [9, 24]. We have encountered a case in which blood retention due to thrombosis appeared as pseudo-lesions on early-phase imaging, potentially leading to misinterpretation [32]. Additionally, elevated blood pool uptake may pose a risk of false-positive findings when evaluating hypervascular lesions.

Although uptake decreased over time in most normal organs, uptake in tumor lesions remained relatively unchanged. Consequently, TBRs increased over time, and the contrast of the tumor lesion was expected to increase in the later phase. However, even the image at 10 min after administration had a TBR of 2.76 in the primary lesions, 1.67 in the lymph node metastases/peritoneal dissemination, and 1.57 overall, which was considered sufficient contrast to detect the lesion. Imaging 10 min after administration may also be considered from the standpoint of examination throughput and patient invasiveness.

This study had several limitations. First, histopathological evaluation of the lesions was not performed in all the cases. Therefore, it was difficult to differentiate peritoneal dissemination from lymph node metastasis, especially in gastrointestinal cancers, and these were analyzed together as one subgroup. Second, as urine was not collected for analysis during the examination, tracer excretion could not be adequately evaluated in this study. Considering the potential correlation between the FAPI uptake in organs and its excretion to bile and urine, further research is necessary to elucidate the relationship between organ uptake and tracer excretion. Third, the sample size was small (32 cases), and consequently, the numbers of analyzable lesions and sex-specific normal organs were further limited. Finaly, this study was conducted at a single institution. Further accumulation of cases is needed to determine the utility of early-phase [^18^F]FAPI-74 PET/CT imaging and to establish the optimal imaging time.

## Conclusion

The uptake of [^18^F]FAPI-74 in most organs gradually decreased over time, except in the mammary glands and nipples. Moreover, uptake due to the excretion increased over time in the gallbladder, and bile duct. In contrast, tumor uptake remained relatively stable, with the highest TBRs observed at 60 min after injection. However, early-phase imaging was considered useful because of its sufficient diagnostic contrast, and it may be more effective in detecting peribiliary lesions.

## Data Availability

Data from this article are available upon reasonable request from the corresponding author.

## Abbreviations

CAFs: Cancer-associated fibroblasts
FAP: Fibroblast activation protein
FAPI: FAP inhibitor
MIP: Maximum intensity projection
SUV: Standardized uptake value
TBR: Tumur-to-blood ratio
